# Robotic Coronary Artery Bypass Grafting: A Narrative Review of Techniques, Evidence, and Future Directions

**DOI:** 10.3390/jcm15176677

**Published:** 2026-08-28

**Authors:** Edgar Aranda-Michel, Matthew M. Duda, Katherine Verdi

**Affiliations:** 1 Division of Cardiac Surgery, Department of Cardiothoracic Surgery, Stanford University, Palo Alto, CA 94304, USA; earandam@stanford.edu (E.A.-M.); mattduda@stanford.edu (M.M.D.); 2 Division of Cardiothoracic Surgery, Department of Surgery, University of Virginia, Charlottesville, VA 22908, USA

**Keywords:** robotic coronary artery bypass, robotic-assisted minimally invasive direct coronary artery bypass (RA-MIDCAB), totally endoscopic coronary artery bypass (TECAB), hybrid coronary revascularization (HCR), learning curve, graft patency

## Abstract

This narrative review summarizes the published literature on robotic coronary revascularization, encompassing robotic-assisted minimally invasive direct coronary artery bypass (RA-MIDCAB), totally endoscopic coronary artery bypass (TECAB), and hybrid coronary revascularization (HCR). These are distinct procedures that differ in operative access, conduit harvesting, use of cardiopulmonary bypass (CPB), and typical patient selection, and are treated as such throughout this review rather than as interchangeable techniques. A structured, non-systematic literature search of PubMed/MEDLINE and major cardiothoracic surgical journals was performed. Articles were selected by the authors based on relevance, methodological quality, and recency. This is explicitly a narrative rather than a systematic review, and no PRISMA methodology was applied. In observational series from experienced centers, RA-MIDCAB and TECAB are associated with low perioperative mortality (0–2.8%), high early left internal thoracic artery (LITA)-to-left anterior descending (LAD) graft patency (>95%), and shorter hospital stay than conventional sternotomy coronary artery bypass grafting (CABG) in selected patients. HCR extends robotic revascularization to selected patients with multivessel and left main disease. However, nearly all of this evidence derives from retrospective, single-center, or registry-based observational studies performed at high-volume expert centers in selected patient populations, and comparative claims against conventional CABG are vulnerable to selection bias and residual confounding. Robotic coronary revascularization is a feasible option that has been reproduced across a growing number of high-volume centers for carefully selected patients, but current evidence does not support broad claims of superiority over conventional CABG, and outcome data remain concentrated among a limited number of expert programs. Prospective multicenter and randomized data, standardized outcome definitions, and structured training pathways are needed to define its long-term role in coronary revascularization.

## 1. Introduction

Coronary artery bypass grafting (CABG) remains the standard revascularization strategy for patients with complex multivessel coronary artery disease (CAD) and left main disease, offering durable long-term survival benefit over percutaneous coronary intervention (PCI) in appropriately selected populations [1,2]. The conventional approach through median sternotomy is effective but carries recognized morbidity, including postoperative pain, risk of deep sternal wound infection, and prolonged recovery. Over the past 25 years, robotic technology has been applied to preserve the durability of left internal thoracic artery (LITA)-to-left anterior descending (LAD) artery grafting while reducing the invasiveness of the surgical approach [3,4].

Contemporary guideline frameworks for coronary revascularization now include the 2024 European Society of Cardiology (ESC) Guidelines for the management of chronic coronary syndromes. These supersede the chronic coronary syndrome-related recommendations of the 2018 ESC/European Association for Cardio-Thoracic Surgery (EACTS) Guidelines on myocardial revascularization as the current primary reference for stable, non-emergent revascularization decision-making. Robotic and minimally invasive techniques are not separately adjudicated in either document and are instead evaluated against the same evidentiary standards applied to conventional CABG [5,6].

To date, many procedural reviews, including the 2024 systematic review and meta-analysis of two decades of outcomes, treat robotic coronary revascularization as a single comparative entity [1]. This review adds to the growing body of secondary literature by explicitly separating procedure-specific evidence regarding RA-MIDCAB, TECAB, and HCR. We further frame each subsequent section around the questions clinicians face: which patients are appropriate candidates, which robotic strategy (or non-robotic comparator) best fits a given anatomy and risk profile, and what level of evidentiary certainty supports each claim? Where relevant, we incorporate the 2024 position paper of the European Society of Cardiology Working Group on Cardiovascular Surgery and the European Association of Percutaneous Cardiovascular Interventions on hybrid coronary revascularization, which addresses procedural sequencing and pharmacological management in greater depth than prior device-focused reviews [7].

Robotic-assisted cardiac surgery was first reported in the late 1990s, when Loulmet, Carpentier, and colleagues performed the first robotically assisted endoscopic coronary anastomosis on an arrested heart in Paris [8]. Falk, Mohr, and colleagues in Leipzig subsequently reported totally endoscopic coronary artery bypass (TECAB) on the arrested heart using the da Vinci Surgical System (Intuitive Surgical, Sunnyvale, CA, USA) [9,10]. These proof-of-concept reports established the technical basis for what later diversified into beating-heart TECAB, robotic-assisted minimally invasive direct coronary artery bypass (RA-MIDCAB), and hybrid coronary revascularization (HCR).

Despite more than two decades of experience, robotic CABG remains a low-volume procedure. Registry analyses of the Society of Thoracic Surgeons (STS) Adult Cardiac Surgery Database estimated that robotic-assisted CABG constituted well under 1% of CABG cases performed in the United States, with only modest growth since [11,12]. Barriers to wider adoption include a prolonged learning curve, high capital and consumable costs, limited structured training pathways, and the absence of large randomized trials directly comparing robotic with conventional CABG [13,14,15].

This review is a narrative synthesis rather than a systematic review or meta-analysis; no formal quality assessment, risk-of-bias scoring, or PRISMA-style article flow was performed. Our objective is to describe the distinct robotic coronary procedures in current use, critically summarize the available evidence on outcomes, and identify gaps that should guide future research.

## 2. Methods

We conducted a structured, non-systematic (narrative) literature search of PubMed/MEDLINE (National Center for Biotechnology Information, Bethesda, MD, USA) and the major cardiothoracic surgical journals (*Annals of Thoracic Surgery*, *Journal of Thoracic and Cardiovascular Surgery*, *European Journal of Cardio-Thoracic Surgery*, *Annals of Cardiothoracic Surgery*, *Innovations*, *Journal of Robotic Surgery*) for English-language publications from January 1998 through June 2026, the date of the final search. Search terms included combinations of “robotic,” “robotic-assisted,” “RA-MIDCAB,” “TECAB,” “hybrid coronary revascularization,” “coronary artery bypass,” and “minimally invasive.” Additional articles were identified by manually reviewing reference lists of relevant papers and prior reviews (snowball search).

Eligible study types included randomized controlled trials, prospective and retrospective cohort studies, propensity-matched analyses, registry and administrative database studies, systematic reviews and meta-analyses, and expert consensus statements. Case reports and non-English publications were excluded. Selection of articles for inclusion and data extraction was performed by the authors. Given the narrative design, no independent duplicate screening, formal inter-rater agreement, or PRISMA flow diagram was employed. Where multiple publications appeared to report overlapping patient cohorts from the same institution, we cross-checked author lists, enrollment periods, and sample sizes to identify and avoid double-counting of outcomes. Overlapping or duplicate citations identified during manuscript preparation were consolidated to a single reference.

Because this is a narrative rather than a systematic review, terms such as “largest series” or “most comprehensive analysis” used throughout this manuscript refer to the largest or most detailed study identified within our non-exhaustive search. These should not be interpreted as claims of completeness across the entire published literature.

## 3. Procedural Taxonomy

Robotic coronary revascularization encompasses several distinct operations that differ substantially in access, technique, and evidence base (Table 1). These procedures should not be treated as interchangeable, and outcomes reported for one technique should not be generalized to another.

## 4. Historical Background and Technological Evolution

The first robotic-assisted endoscopic coronary anastomosis was performed on an arrested heart by Loulmet, Carpentier, and colleagues at the Hôpital Broussais in Paris in 1998, using an early prototype robotic arm system [8]. In the same year, Falk, Mohr, and the Leipzig group performed the first TECAB on the arrested heart using the da Vinci Surgical System, demonstrating that endoscopic coronary anastomoses could be fashioned robotically with acceptable early patency [9]. Mohr and colleagues subsequently reported a larger 148-patient series of computer-enhanced robotic cardiac procedures that further supported the platform’s technical feasibility [10].

The following decade saw expansion to the beating heart. Kappert and colleagues described closed-chest, beating-heart TECAB using CO_2_ insufflation of the left hemithorax and dedicated endostabilizers [29]. Balkhy and colleagues at the University of Chicago subsequently reported progressively larger beating-heart TECAB series using automated anastomotic connectors, culminating in a single-institution 10-year experience of 874 patients—the largest published TECAB series identified in our search [24,30,31,32,33]. In parallel, RA-MIDCAB—endoscopic LITA harvesting followed by hand-sewn anastomosis through a 3–5 cm left anterior thoracotomy—emerged as a technically simpler, more widely adopted alternative [3,4].

HCR was formally described by Katz and colleagues in a multicenter series of 27 patients across seven centers, reporting 96.3% LITA anastomotic patency at three months [34]. Srivastava and colleagues at the University of Maryland subsequently classified staged HCR sequences (surgery-first versus PCI-first) and reported that, within their observational cohort, sequence did not appear to significantly influence clinical outcomes—a finding that has not been confirmed in a randomized comparison [25]. Since then, robotic coronary surgery has expanded to include multivessel and bilateral internal thoracic artery (BITA) grafting, application in left main disease, redo operations, and single-center series with up to a decade of follow-up [13,20,21,24,35].

## 5. Patient Selection

Patient selection is central to the safety and success of robotic coronary surgery and warrants a structured algorithmic approach, integrating coronary anatomy, thoracic geometry, pulmonary function, and cardiac status through a dedicated Heart Team evaluation [16].

### 5.1. Favorable Characteristics

Favorable candidates typically have a non-calcified, epicardial (non-intramyocardial) LAD target ≥ 1.5 mm in diameter with a suitable distal landing zone, preserved thoracic working space on preoperative CT (anteroposterior-to-transverse diameter ratio > 0.45), preserved left ventricular function, and adequate pulmonary reserve to tolerate one-lung ventilation (FEV_1_ and DLCO generally ≥40% predicted) [16]. These specific numeric thresholds originate from single-center or expert-derived technical protocols rather than externally validated, outcome-linked cutoffs tested across multiple cohorts; they are best regarded as center-specific technical heuristics that inform, rather than replace, individualized multidisciplinary assessment.

### 5.2. Unfavorable Characteristics and Contraindications

Absolute contraindications include inability to tolerate one-lung ventilation, hemodynamic instability or need for emergency surgery, prior pneumonectomy, and absence of a technically approachable anastomotic target [16,36]. Relative contraindications—prior left thoracotomy or pleurodesis, severe pleural adhesions, intramyocardial LAD, severe coronary calcification, morbid obesity, chest wall deformity, significant peripheral or aortic calcification precluding femoral cannulation (relevant to arrested-heart TECAB), severely reduced ejection fraction, moderate-to-severe pulmonary hypertension, and need for concomitant valve or aortic surgery not addressable robotically—require individualized assessment rather than automatic exclusion. Experienced centers have safely extended candidacy to selected patients with several of these features, but this experience should not be generalized to lower-volume programs [24].

Patient-level factors that influence candidacy but are addressed inconsistently across the literature include frailty, sex, diabetes mellitus, chronic kidney disease, obesity, diffuse (rather than focal) coronary disease, and small-caliber target vessel anatomy. Robotic-specific outcome data stratified by these characteristics remain limited, and eligibility decisions for such patients should rely on multidisciplinary Heart Team judgment rather than the general selection criteria above. Sex-specific differences in coronary anatomy, plaque distribution, and presentation patterns in particular warrant consideration during candidacy assessment, as women are more likely to have non-obstructive or diffuse disease phenotypes that may affect suitability for a focused robotic approach [37].

Qureshi and Ruel similarly emphasize mandatory multidisciplinary Heart Team review as a foundational requirement for safe multivessel minimally invasive coronary surgery [38]. In line with this principle, we recommend that this team include an experienced robotic cardiac surgeon, an interventional cardiologist, and a cardiac anesthesiologist familiar with one-lung ventilation and capnothorax physiology for every candidate considered for minimally invasive multivessel coronary surgery, a recommendation endorsed throughout this review.

### 5.3. Expanding Indications at Experienced Centers

At high-volume programs, multivessel disease has been addressed through RA-MIDCAB with BITA grafting, TECAB, or HCR, depending on coronary anatomy and institutional expertise [21,39]. In small single-center series, surgery-first HCR has been reported as feasible in selected patients with left main disease. A reverse-HCR strategy (PCI first, robotic surgery second) has likewise been described in small cohorts. Both approaches should currently be regarded as feasible in expert hands rather than as established general strategies, given the small sample sizes, absence of comparator arms, and lack of information on left main lesion complexity, SYNTAX score, and antiplatelet management in these reports [40,41]. Redo coronary surgery via a robotic left thoracic approach has been reported as feasible in patients with a virgin left hemithorax in a small dedicated series, representing an emerging but not yet well-validated application [19]. Case volume concentration is itself a component of safe patient selection. Dedicated learning curve data indicate that stable outcomes require substantial cumulative institutional experience, supporting referral of complex candidates to established programs [17].

## 6. Operative Techniques

### 6.1. Single-Vessel RA-MIDCAB

RA-MIDCAB uses the da Vinci Surgical System for endoscopic LITA harvesting, with the coronary anastomosis performed under direct vision through a 3–5 cm left anterior thoracotomy on the beating or transiently stabilized heart. Port placement typically involves three or four robotic trocars in the left hemithorax with CO_2_ insufflation and one-lung ventilation. The LITA is harvested as a skeletonized or pedicled conduit, and the anastomosis is constructed with 7-0 polypropylene sutures using a mechanical tissue stabilizer [16,22,42]. This single-vessel approach is limited to LAD revascularization and is not a substitute for complete revascularization in patients with significant disease in other territories.

### 6.2. Multivessel RA-MIDCAB

Multivessel RA-MIDCAB using in situ BITA grafting has been reported in a single-center series of 221 patients, with 12-month graft patency of 97.1% and survival of 98.5%—the largest contemporary multivessel RA-MIDCAB report identified in our search [21]. This approach permits grafting of the LAD, diagonal, and obtuse marginal territories through a single left-sided access. Reported outcomes in small comparative series appear broadly similar to staged HCR, though no adequately powered head-to-head comparison exists [20,23,43].

### 6.3. Totally Endoscopic Coronary Artery Bypass (TECAB)

TECAB is considered the most technically demanding form of robotic coronary surgery, in which both LITA harvesting and coronary anastomosis are performed endoscopically without a thoracotomy. Arrested-heart TECAB uses femoral cardiopulmonary bypass (CPB) with endoaortic balloon occlusion, while beating-heart TECAB avoids bypass entirely, using robotic endostabilizers and, in some series, automated anastomotic connectors [3,4,44]. These are mechanically and physiologically distinct approaches with different risk profiles, and outcomes should not be pooled without distinguishing them. In the largest single-center beating-heart TECAB series identified (874 patients, 10-year experience), perioperative mortality was 0.9%, early graft patency 97%, and freedom from major adverse cardiac and cerebrovascular events (MACCE) 93% during a mean follow-up of approximately 48 months, with the longest observation extending to 10.6 years. These findings originate from one high-volume expert center and have not yet been replicated at other institutions [24].

BITA TECAB (BITA grafting performed totally endoscopically) has been proposed as a means of achieving total arterial revascularization without sternotomy and without the wound-related morbidity of open BITA harvesting [13,45]. At present, BITA TECAB should be regarded as a technically feasible extension of established BITA principles to a robotic platform, rather than as an approach with its own dedicated long-term comparative survival data.

### 6.4. Hybrid Coronary Revascularization (HCR)

HCR combines robotic LITA-LAD grafting with PCI of non-LAD vessels, with the goal of extending robotic revascularization to selected patients with multivessel disease. HCR may be performed in a staged (surgery-first or PCI-first) or simultaneous (“one-stop”) fashion.

Surgery-first staged HCR allows confirmation of LITA-LAD graft patency before proceeding to PCI and protects the LAD territory during the interval. However, untreated non-LAD lesions may experience ongoing ischemia, and delayed initiation of dual antiplatelet therapy (DAPT) may be necessary in the event of post-operative bleeding complications. PCI-first staged HCR promptly addresses unstable non-LAD lesions but may expose the patient to surgery in the setting of recent DAPT. DAPT continuation likely increases bleeding and transfusion risk, while perioperative DAPT interruption increases the risk of stent thrombosis. Simultaneous HCR permits immediate completion angiography and graft revision if needed but requires a hybrid operating room and coordinated multidisciplinary team in addition to possibly greater resource utilization, procedural time, and radiation and contrast exposure [25,28].

The 2024 ESC/European Association of Percutaneous Cardiovascular Interventions (EAPCI) hybrid coronary revascularization position paper provides more detailed guidance on procedural sequencing. According to these groups, the interval between surgical and percutaneous components should be individualized based on bleeding risk, ischemic burden, and clinical stability. Renal contrast burden becomes an additional consideration when staged angiography or PCI closely follows cardiac surgery in patients with reduced renal reserve [7]. Device selection for the non-LAD percutaneous component is typically a drug-eluting stent, though drug-coated balloon angioplasty has emerged as an alternative for selected non-LAD lesions in the broader PCI literature; dedicated evidence evaluating drug-coated balloons specifically within an HCR treatment strategy is not yet available [46].

## 7. Perioperative and Long-Term Outcomes

### 7.1. Evidence Hierarchy

Before summarizing outcomes, it is important to characterize the quality of the underlying evidence. The large majority of published data on robotic CABG and HCR derive from retrospective, single-center, observational series conducted at high-volume expert institutions; propensity-matched registry analyses; and meta-analyses that are themselves dominated by observational studies. Such studies are often subject to residual confounding, center clustering, propensity score diagnostics, competing risks, and overlapping institutional cohorts among other limitations. Randomized evidence is limited to comparisons of HCR versus conventional CABG versus multivessel PCI (HREVS, MERGING pilot trial) and does not isolate the incremental effect of the robotic component itself [26,27]. Expert consensus statements and technical reviews, while valuable for procedural guidance, do not constitute outcome data and are not cited as such in this review. Throughout this section, we specify the study design underlying each reported finding and avoid presenting observational associations as proof of causal superiority (Table 2).

### 7.2. Mortality and Major Morbidity

Contemporary robotic CABG series report low operative mortality across technique variants. In the largest meta-analysis identified in our search, pooling two decades of predominantly observational reported outcomes, Hwang and colleagues described favorable short-term mortality and hospital length of stay (LOS) for both RA-MIDCAB and TECAB, while noting substantial heterogeneity across included studies [1]. A complementary meta-analysis by Wilson-Smith and colleagues reported similar findings, again drawing predominantly on observational data [2,54,55,56]. Individual large single-center series report 30-day mortality of 0.6% in a 1000-case RA-MIDCAB series [17], 0.9% in the 874-patient TECAB series [24], and 0% in small initial-experience cohorts at newly adopting centers [18].

Robotic CABG has been associated with lower rates of blood transfusion, deep sternal wound infection, atrial fibrillation (AF), and acute kidney injury (AKI) compared with conventional CABG in multiple observational and propensity-matched comparisons [24,39,41,50,51]. These associations should be interpreted cautiously; patients selected for robotic approaches are frequently younger, less frail, less calcified, less obese, and less likely to require urgent surgery or complex multivessel grafting than those undergoing conventional CABG. Retrospective matching cannot fully eliminate this selection bias. In a large propensity-matched TriNetX (TriNetX LLC, Cambridge, MA, USA) analysis of 1796 robotic versus 249,580 non-robotic CABG patients, robotic CABG was associated with similar long-term survival and lower rates of stroke, but a higher rate of repeat revascularization at extended follow-up—an association, not a demonstration of causal effect [48].

### 7.3. Length of Stay and Recovery

Reduction in hospital LOS is among the most consistently reported associations with robotic CABG. Across major series, reported mean or median hospital stay after RA-MIDCAB ranges from approximately 3.9 to 5 days, compared with 5–7 days reported for conventional CABG in the same studies. However, LOS comparisons are confounded by differences in institutional discharge practice, use of enhanced recovery after surgery (ERAS) protocols, and healthcare system. Cross-study or cross-country comparisons should be interpreted cautiously [24,50]. In the Netherlands nationwide registry, RA-MIDCAB and HCR were associated with shorter intensive care unit (ICU) stay and earlier discharge compared with conventional CABG in an observational, non-randomized comparison [49].

### 7.4. Graft Patency and Completeness of Revascularization

Graft patency should be distinguished by assessment timing and method: early technical patency, typically assessed by intraoperative flow measurement or early postoperative angiography (either invasive or CT); midterm patency, assessed by systematic angiographic surveillance or symptom-driven (clinically triggered) evaluation; and long-term patency, also assessed by angiographic surveillance or symptom-driven evaluation. Most available robotic CABG patency data derive from smaller-scale and often symptom-driven assessments rather than protocol-mandated angiographic follow-up, which limits confidence in reported long-term patency figures and their comparability to conventional open LITA harvest [57].

In a systematic review by Kitahara and colleagues encompassing RA-MIDCAB and TECAB series, early LITA-LAD patency was reported at 97.7%, declining to 96.1% at midterm and 93.2% at longer-term follow-up in the subset of patients undergoing angiographic assessment. The authors noted these figures are broadly comparable to historical open LITA-LAD series but caution that direct head-to-head angiographic comparison trials are lacking [57]. A 500-case TECAB series reported similar angiographic patency, and the 874-patient TECAB series described in Section 6.3 reported comparable intermediate-term patency (97%). Both are single-center observational series without a concurrent open-CABG comparator arm [24,30,58].

The evidence base for graft patency is dominated by LITA-LAD grafts. Data for radial artery, saphenous vein, sequential grafting configurations, and non-LAD targets performed robotically remain limited. Anatomical completeness of revascularization (number of distal anastomoses achieved) should also be distinguished from functional completeness (adequacy of perfusion to all ischemic territories). The literature rarely reports both metrics together, and the feasibility of grafting diffusely diseased, heavily calcified, intramyocardial, or small-caliber lateral and inferior wall targets robotically remains incompletely characterized.

### 7.5. Long-Term Comparative Outcomes

Kofler and colleagues published a propensity-matched comparison of 134 robotic versus 134 conventional CABG patients (mean follow-up 6.6 years), reporting statistically similar MACCE-free and overall survival between groups (MACCE component definitions vary across studies cited in this review; see Section 13). As a single-center, propensity-matched (non-randomized) comparison, residual confounding by indication cannot be excluded [47]. Dokollari and colleagues reported the largest single-institution longitudinal series identified (2280 consecutive robotic-assisted CABG patients, 2005–2021), describing an increase in HCR utilization from 25.5% to 48.4% over the study period and improved outcomes with increasing institutional experience—an observation consistent with a learning curve or center experience effect rather than an isolated technique effect [35].

The TriNetX propensity-matched analysis (1796 robotic versus 249,580 non-robotic CABG patients) similarly reported comparable long-term survival, lower stroke rates, and higher long-term repeat revascularization associated with robotic CABG [48]. The higher repeat revascularization rate likely reflects, at least in part, the inclusion of HCR patients (in whom PCI in-stent restenosis (ISR) contributes to repeat intervention) and possible differences in patient selection between robotic and conventional cohorts. The administrative/claims nature of the TriNetX database does not permit adjustment for angiographic or anatomical detail.

A nationwide Netherlands registry study by de Jong and colleagues provided population-level, non-randomized observational data suggesting that robotic coronary surgery can be performed safely outside high-volume academic referral centers; however, outcomes at lower-volume centers were not separately reported in sufficient detail to confirm generalizability [49]. A propensity-matched comparison by Shekar and colleagues reported lower postoperative AF in a robotic cohort, with a cumulative sum (CUSUM) learning curve analysis suggesting proficiency by the 86th case in that single series—a center-specific finding that should not be extrapolated as a universal training benchmark [59].

## 8. Hybrid Coronary Revascularization

### 8.1. Observational Evidence

Multiple meta-analyses, dominated by observational comparisons, have evaluated HCR outcomes against conventional CABG. A meta-analysis by Sardar and colleagues reported that HCR was associated with lower transfusion and infection rates, shorter hospital stay, and comparable short-term MACCE versus CABG, but higher long-term target vessel revascularization (TVR), attributed by the authors chiefly to PCI ISR rather than surgical graft failure [50]. A meta-analysis by Nagraj and colleagues (14 studies, 4226 patients) reported statistically similar 5-year mortality and long-term MACCE between HCR and CABG, with HCR associated with shorter hospital stay but higher repeat revascularization [51,60,61]. A 2025 meta-analysis by Schuering and colleagues (32 studies, 2048 patients) reported a favorable perioperative safety profile for robotic HCR, again based predominantly on observational comparative data [62]. As noted in Section 7.5, Dokollari and colleagues reported a substantial increase in HCR utilization at their center over the study period, an institutional trend rather than a comparative outcome finding [35].

### 8.2. Randomized Trial Evidence

The HREVS trial randomized 155 patients with multivessel CAD to CABG, HCR, or multivessel PCI. At 12 months, residual myocardial ischemia by SPECT was statistically non-inferior for HCR versus CABG (5% versus 5%), and MACCE rates were similar across the three arms (12% CABG, 13.4% HCR, 13.2% PCI; *p* = 0.83). MACCE component definitions vary across the studies cited in this review and are addressed in Section 13 [26]. The MERGING pilot randomized trial allocated 60 patients with complex triple-vessel CAD to HCR or CABG in a 2:1 ratio. The primary composite endpoint (death, myocardial infarction, stroke, or unplanned revascularization) occurred in 19.3% of HCR versus 5.9% of CABG patients, driven primarily by unplanned revascularization in the HCR arm. As a pilot trial with a small sample size, MERGING was not powered to definitively establish superiority of either strategy, and this event-rate difference warrants confirmation in a larger trial. Notably, neither HREVS nor MERGING specifically isolated robotic assistance as a variable. Both compared a hybrid revascularization strategy against CABG or PCI broadly. Their findings characterize hybrid revascularization as a strategy rather than the comparative effectiveness of robotic technology itself and should not be conflated with evidence for or against the robotic component specifically [27].

Balkhy and colleagues reported a propensity-matched analysis of 418 intent-to-treat HCR patients. Among the approximately 20% in whom the planned PCI component could not be completed, omission of PCI was not associated with a statistically significant difference in midterm survival or major adverse events [63]. This finding should be interpreted cautiously. Failure to demonstrate a significant difference in a moderate-sized, non-randomized cohort with a relatively short follow-up does not establish that incomplete revascularization is safe or equivalent to planned complete HCR, and the sample size, event rate, and potential for selection bias (patients in whom PCI failed may differ systematically from those in whom it succeeded) limit the strength of this conclusion. Randomized and comparative studies of HCR are summarized in Table 3.

### 8.3. Bilateral Internal Thoracic Artery (BITA) Grafting

BITA TECAB has been proposed as an approach to total arterial revascularization. Support for BITA TECAB is sometimes attributed to long-term survival data from the Arterial Revascularization Trial (ART) and SYNTAX trials. Yet, the ART reported by Taggart and colleagues at 10 years did not demonstrate a statistically significant intention-to-treat survival advantage of bilateral over single internal thoracic artery grafting [45]. Interpretation of ART was complicated by substantial crossover between study arms and frequent concomitant use of radial artery grafting. Any suggestion of a demonstrated survival benefit of BITA grafting in ART should therefore be limited to as-treated or per-protocol subgroup analyses, which carry weaker causal inference than the intention-to-treat result.

The biological and surgical rationale for BITA grafting—more complete arterial revascularization and avoidance of vein graft attrition—remains plausible and is supported by some observational registry data, but it should not be presented as conclusively proven by the cited randomized evidence [13]. Accordingly, BITA TECAB should be described as a technically feasible extension of arterial revascularization principles with observational supporting data, rather than as an approach with a demonstrated randomized survival advantage.

### 8.4. Simultaneous Hybrid Revascularization

The simultaneous (“one-stop”) HCR strategy, performing LITA-LAD bypass and PCI in the same session in a hybrid operating room, offers potential logistical and antiplatelet timing advantages over staged approaches, at the potential cost of greater procedural complexity and resource utilization. Zhao and colleagues reported that routine intraoperative completion angiography during one-stop HCR identified graft abnormalities in a subset of patients and permitted immediate revision, in a single-center observational series [64]. Kon and colleagues reported that simultaneous HCR was associated with reduced postoperative morbidity compared with off-pump CABG in a non-randomized comparison [28]. The 2018 European Society of Cardiology/European Association for Cardio-Thoracic Surgery (ESC/EACTS) guidelines assigned a Class IIb (level of evidence C) recommendation for HCR in selected patients with multivessel CAD at experienced centers. This Class IIb designation should be understood as indicating that the procedure may be considered in selected circumstances, not as an endorsement of broad or first-line use [5].

## 9. Learning Curve and Program Development

The learning curve is among the most frequently cited barriers to broad adoption of robotic CABG. Learning curve terminology is used inconsistently across the literature, and we distinguish the following endpoints where possible: basic console familiarity (ability to operate the robotic system), technical feasibility (ability to complete the procedure without conversion to sternotomy), procedural competency (stabilization of operative time and conversion rate at an acceptable threshold), proficiency (consistent achievement of low complication rates), and mastery (outcomes that plateau at their best achievable level, including for more complex configurations such as multivessel or BITA grafting).

Patrick and colleagues analyzed 1195 robotic CABG procedures performed by 114 surgeons in the STS database, reporting that operative time and conversion outcomes stabilized after approximately the 10th procedure per surgeon—a competency threshold that reflects a minimum case volume rather than the full trajectory of skill acquisition. This study did not separately report proficiency or mastery endpoints [53]. Jonsson and colleagues analyzed 1000 consecutive RA-MIDCAB procedures at a single center (Emory University), reporting that mean procedure time decreased from 195 min in the first 500 cases to 176 min in the second 500 cases; conversion to sternotomy fell from 4.4% to 1.6%. Overall 30-day mortality was 0.6%, and graft patency was 97.2% among 505 angiographically assessed patients. The authors defined mastery as sustained outcomes at or below these thresholds, concluding that 250 to 500 cases were required—substantially exceeding typical credentialing volume requirements at most programs and representing surgeon-level, single-procedure (RA-MIDCAB) learning at one high-volume center [17]. Rosati and colleagues reported that patient complexity did not adversely affect safety during the early RA-MIDCAB learning curve at a newly adopting Italian center, a center-level finding on program initiation rather than an individual surgeon mastery endpoint [65].

None of the available learning curve studies isolate the influence of prior minimally invasive or off-pump CABG experience, structured proctoring, progressive case selection evolution, or team-level learning among anesthesiology, nursing, perfusion, and interventional cardiology staff, all of which likely affect the trajectory described above. A survey of 75 coronary surgeons at the 2023 STS annual conference identified insufficient skill transfer, limited referral support, and absence of long-term outcome data as the three most frequently cited barriers to initiating or expanding a robotic CABG program [15]. The principal learning curve studies in robotic CABG are summarized in Table 4.

## 10. Cost and Resource Utilization

Economic analyses of robotic CABG must weigh higher capital and consumable costs of the robotic platform against potential offsets from shorter hospital stay, reduced blood product use, lower wound complication rates, and faster return to activity. Comparative cost data remain limited, and the comparison between TECAB and RA-MIDCAB reported below is not equivalent to a direct comparison between robotic CABG broadly and conventional CABG.

Pasrija and colleagues reported a direct cost comparison between TECAB and RA-MIDCAB, finding comparable short-term clinical outcomes but significantly higher hospital costs for TECAB ($33,769 versus $22,679, *p* < 0.001), attributed to longer operative time and higher consumable costs. This study did not include a conventional CABG comparator arm [52]. Dokollari and colleagues, in a 1173-patient propensity-matched analysis, reported lower mean direct and total cost for robotic-assisted CABG versus conventional CABG ($25,300 versus $33,400), attributed to shorter LOS and reduced transfusion requirements. This source was presented as a meeting abstract at the time of our search rather than a full peer-reviewed economic analysis and should be regarded as preliminary pending full publication [66].

Comprehensive economic evaluation of robotic CABG should ideally also account for capital acquisition and maintenance costs, disposable and instrument costs, robot utilization across specialties sharing the same platform, operating room and hybrid room time, PCI and drug-eluting stent costs in HCR, readmissions, repeat revascularization, rehabilitation, return to work, societal costs, and geographic variation in reimbursement and amortization assumptions; few, if any, published studies address this full scope. Zhuli and colleagues, analyzing 10,543 robotic cardiac surgeries (not limited to CABG), reported better outcomes at higher-volume centers, with costs approaching those of non-robotic equivalents in that cohort, a volume–cost relationship also reported in robotic mitral valve repair [67,68]. Given the limitations above, the notion that total cost of robotic CABG is comparable to or lower than conventional CABG at high-volume centers should be regarded as hypothesis-generating rather than established, pending more complete peer-reviewed comparative economic studies.

## 11. Anesthetic and Perioperative Management

Robotic CABG, particularly TECAB, imposes anesthetic challenges that demand specialized expertise and center-specific protocols [36,69,70]. Left-sided robotic coronary procedures generally require isolation of the left lung to permit surgical access, commonly achieved with a left-sided double-lumen endotracheal tube or a bronchial blocker positioned to collapse the left lung while ventilating the right lung. Specific device and technique vary by center and anesthesiologist preference. Additional key physiological considerations include CO_2_ capnothorax from left hemithorax insufflation (typically 8–12 mmHg), hemodynamic effects of mediastinal compression during cardiac manipulation, and limited surgical access for emergent intraoperative rescue.

Intraoperative monitoring strategies, including transesophageal echocardiography (TEE), pulmonary artery catheterization, and cerebral near-infrared spectroscopy, are used selectively based on center-specific protocols and patient risk factors rather than being universally mandatory. TEE is used most consistently, while pulmonary artery catheterization and cerebral oximetry are employed more variably [36]. External defibrillator pad positioning, arterial and central venous access, detection and management of tension capnothorax, and availability of a rapid-access emergency conversion to sternotomy and CPB protocol are essential components of preparation regardless of specific monitoring choices [36,69]. Deshpande and colleagues provided an earlier foundational review specifically addressing TECAB anesthesia and the physiology of one-lung ventilation in the context of capnothorax [69].

Holmes and colleagues published a 2025 review of anesthesia for minimally invasive CABG covering the perioperative pathway from preoperative risk stratification through ERAS protocols, including multimodal analgesia (regional blocks such as erector spinae or serratus anterior plane blocks where used), early extubation, and same-day or next-day mobilization strategies relevant to realizing the recovery benefits associated with the robotic approach [70].

## 12. Conversion to Sternotomy and Emergency Management

Conversion to sternotomy is a major safety endpoint in robotic coronary surgery; yet, published series report highly variable conversion rates and rarely provide detailed cause-specific breakdowns. Reported causes of conversion include bleeding, conduit injury, inadequate target exposure, hemodynamic instability, graft dysfunction, and equipment failure. Series differ in whether conversions are classified as elective (e.g., for exposure) or emergency (e.g., for hemodynamic collapse), which affects interpretation of reported rates. In the largest single-center RA-MIDCAB learning curve series, conversion fell from 4.4% in the first 500 cases to 1.6% in the second 500 cases as institutional experience accumulated [17].

Robotic programs should maintain an established, rehearsed emergency conversion protocol, including rapid access to CPB, pre-positioned emergency instrumentation, and defined roles for emergency undocking of the robotic system, given that time to sternotomy directly affects the ability to manage intraoperative hemodynamic instability. The published literature we reviewed does not provide a systematic, cross-study analysis of the relationship between conversion and downstream morbidity or mortality, representing a notable gap for future study.

## 13. Standardization of Outcome Definitions

Outcomes reported across the robotic CABG literature—mortality, MACCE, repeat revascularization, graft patency, stroke, AKI, AF, wound infection, and hospital LOS—are not uniformly defined among studies. Where specified, some studies apply Society of Thoracic Surgeons definitions, Academic Research Consortium criteria, or the Universal Definition of Myocardial Infarction. Many studies, however, do not explicitly report which definitions were used, limiting cross-study comparability and pooling in meta-analyses. MACCE composition (which specific events are included) varies across studies cited in this review and should not be assumed identical; where possible, we have specified the individual components when describing MACCE outcomes.

Repeat revascularization should be further disaggregated into target lesion revascularization, TVR, repeat PCI, repeat CABG, graft failure, and disease progression in previously untreated (non-grafted, non-stented) vessels. Several HCR studies cited in this review attribute the majority of repeat revascularization to PCI ISR, but this attribution is not always confirmed by angiographic follow-up in the primary source, and disease progression in untreated territories is a plausible alternative or contributing explanation that is not routinely excluded.

## 14. Special Topics

### 14.1. Multivessel Disease and Left Main Coronary Artery Disease

Zhou and colleagues compared multivessel RA-MIDCAB (105 patients) with HCR (81 patients) at a single center, reporting comparable outcomes including 0% perioperative mortality in both groups. As a retrospective, non-randomized single-center comparison, this finding should not be read as establishing equivalence between the two strategies [20]. Fu and colleagues reported the largest multivessel BITA RA-MIDCAB series identified (221 patients), with 12-month graft patency of 97.1% and survival of 98.5% [21]. In the literature, small series describing robotic MIDCAB combined with PCI for left main disease report encouraging short-term results, but the current evidence—limited by small sample size, absence of a comparator arm, and incomplete reporting of left main lesion anatomy, SYNTAX score, and antiplatelet strategy—supports this approach as feasible only in carefully selected patients at highly experienced centers, not as an established general strategy for left main disease [40].

### 14.2. Redo Coronary Surgery

Redo coronary revascularization through repeat median sternotomy carries substantially higher morbidity than primary CABG because of mediastinal adhesions and the risk to patent grafts during re-entry. A robotic left thoracic approach, avoiding resternotomy, is an attractive alternative in selected patients with a virgin left hemithorax. Yamashita and colleagues published the first dedicated series of robotic-assisted redo CABG, reporting encouraging early operative outcomes. Given the small size and single-center nature of this series and the inherently higher risk of the redo population, broader applicability remains to be established [19].

### 14.3. Volume–Outcome Relationships and Training

Volume–outcome relationships are consistently reported for robotic CABG. Zhuli and colleagues’ national cohort analysis of 10,543 robotic cardiac surgeries reported significantly better outcomes at higher-volume centers [67]. The expert consensus statement from Bonatti and colleagues offers practical, expert-opinion-based (rather than trial-based) recommendations for program development, including minimum volume targets, proctoring protocols, simulation training, and structured credentialing pathways [71]. Qureshi and Ruel similarly recommend that minimally invasive multivessel CABG be concentrated at centers with established expertise and a dedicated multidisciplinary team [38]. Bonatti and colleagues described structured RA-MIDCAB training incorporating preclinical dry-lab and wet-lab simulation, mentored proctoring, and progressive case complexity escalation [71]; survey data from AlJamal and colleagues indicate that absence of structured training pathways and formal proctoring remains among the most frequently cited barriers to initiating a new robotic CABG program [15].

### 14.4. Practical Clinical Implications

For practicing clinicians, the evidence summarized above suggests that robotic coronary revascularization is most likely to benefit patients with focal, non-calcified, single-vessel LAD disease who are anatomically suitable for a minimally invasive approach and who are treated at experienced, high-volume centers with established robotic programs. In this population, the approach offers a plausible reduction in perioperative morbidity and length of stay without an established sacrifice in graft patency. For patients with multivessel disease, current evidence does not support a uniform preference for RA-MIDCAB, TECAB, or HCR over conventional CABG. Selection among these options should be individualized through multidisciplinary Heart Team discussion incorporating coronary anatomy, comorbidity burden, and local expertise rather than applied as a default strategy. Outside of high-volume expert programs, the comparative safety and effectiveness of robotic approaches are less well established, and lower-volume centers considering program development should weigh the resource requirements and learning curve considerations discussed in Section 9 and Section 14.3 before extending robotic techniques to complex anatomy.

## 15. Limitations of the Current Evidence Base

The existing literature on robotic CABG has several important limitations. The overwhelming majority of outcome data derive from single-center, retrospective, observational series conducted at high-volume referral centers, introducing selection bias and limiting generalizability to average-volume programs [1,2,3,4]. No large, multicenter, randomized trial directly compares robotic with conventional CABG. Available randomized data (HREVS, MERGING) address HCR versus CABG versus multivessel PCI without isolating the robotic component itself [26,27]. Reporting heterogeneity—particularly in defining HCR staging, outcome endpoints, and angiographic follow-up protocols—limits pooled analysis and cross-study comparison.

Long-term outcome data beyond 5–10 years are available from only a small number of institutional series, and population-level registry studies have only recently reached sample sizes sufficient for meaningful comparative effectiveness analysis [25,35,72]. Cost data are limited by institutional variability in accounting methodology, absence of standardized cost-capture frameworks, and frequent omission of societal costs such as productivity loss and rehabilitation expense [52,66]. Systematic angiographic surveillance data, as opposed to symptom-driven graft assessment, remain scarce, and data for non-LITA-LAD grafts performed robotically are particularly limited.

## 16. Future Directions

The most important unmet research need is a prospectively designed, adequately powered, multicenter randomized trial comparing robotic CABG with conventional CABG using appropriate endpoints. Composite endpoints that combine MACCE and graft patency into a single primary endpoint are methodologically problematic. MACCE is ascertained through clinical event adjudication, while graft patency is ascertained through imaging. The two are subject to different missing-data mechanisms, and they do not necessarily move together. Future trials should therefore prespecify MACCE and graft patency as separate, clearly defined endpoints rather than pooling them into a single composite. A well-designed trial should incorporate standardized outcome definitions: protocol-mandated, blinded core-laboratory-adjudicated CT angiographic or angiographic follow-up for patency; a dedicated 30-day safety endpoint; long-term MACCE reported with its individual components; repeat revascularization disaggregated by mechanism; and secondary endpoints assessing patient-reported outcomes, quality of life, return to work, healthcare costs, treatment crossovers, and centre volume effects on outcomes.

Several technologies are frequently discussed as potential enablers of broader robotic CABG adoption. These exist at varying stages of maturity and should be distinguished accordingly. Currently available technologies include existing robotic platforms with three-dimensional visualization and wristed instrumentation. Technologies in clinical evaluation or early adoption include automated anastomotic connectors (already used in some beating-heart TECAB series) and enhanced intraoperative imaging integration [16,73]. Artificial intelligence-assisted operative guidance, automated anastomotic quality assessment, and robotic telestration for remote proctoring remain largely in preclinical or early investigational stages at the time of our search [74]. The suggestion that these emerging technologies may substantially reduce the learning curve and broaden accessibility is a plausible hypothesis based on their intended function, but it is not yet supported by prospective clinical evidence. We present it here explicitly as a hypothesis for future investigation rather than as an established conclusion.

## 17. Conclusions

Robotic coronary revascularization comprises a heterogeneous group of procedures ranging from single-vessel RA-MIDCAB to multivessel TECAB and HCR. In selected patients treated at experienced centers, these approaches are associated with low perioperative mortality, high early LITA-LAD graft patency, shorter recovery, and avoidance of median sternotomy. However, most available evidence derives from retrospective, single-center, observational studies, and comparisons with conventional CABG remain vulnerable to selection bias and residual confounding. Equivalent survival in propensity-matched analyses should not be interpreted as proof of equivalence between surgical strategies.

The learning curve to reach an early process competency threshold—stabilization of operative time and conversion rate, not evidence of safe independent proficiency or mastery—spans approximately 10 cases per the largest available multicenter registry analysis, while mastery of single-vessel RA-MIDCAB has been reported to require 250 to 500 cases in one high-volume single-center series. These figures should not be conflated, and comparable mastery data are not yet available for multivessel RA-MIDCAB, TECAB, or HCR [17,53]. Repeat revascularization is higher in some HCR analyses, attributable at least in part to PCI-related ISR. Cost-effectiveness data remain preliminary, and large multicenter randomized comparisons with conventional CABG remain absent.

The role of robotic techniques in complex multivessel and left main coronary artery disease, their cost-effectiveness, and their long-term comparative durability require further evaluation in prospective multicenter studies and randomized trials. Standardized definitions, structured training pathways, careful patient selection, and systematic long-term follow-up will be essential for broader and safer adoption of robotic coronary revascularization.

## Figures and Tables

**Table 1:** Taxonomy of Robotic Coronary Revascularization Procedures.

Procedure	Access	Conduit Harvest	Anastomosis Method	CPB Use	Typical Grafts	Patient Selection	Key Limitation	Evidence Level (Supporting References)
RA-MIDCAB (single-vessel)	3–5 cm left anterior thoracotomy	Endoscopic robotic LITA harvest	Hand-sewn, direct vision, beating heart	None required, optional pump-assist	1 (LITA-LAD)	Isolated LAD disease	Limited to LAD territory unless combined with HCR/multivessel	Retrospective cohorts, single-center series [16,17,18,19]
Multivessel RA-MIDCAB (BITA)	Left anterior thoracotomy	Endoscopic BITA harvest	Hand-sewn through thoracotomy	None required, optional pump-assist	2–3 (LITA/RITA, composite or in situ)	Multivessel disease, favorable lateral/inferior targets	Technically demanding; limited long-term data	Single/multi-center retrospective series [20,21,22,23]
Arrested-Heart TECAB	Fully endoscopic (3–4 ports)	Endoscopic robotic LITA harvest	Robotic, endoscopic, under cardioplegic arrest	Femoral CPB + endoaortic balloon occlusion	1–2	Favorable anatomy, tolerant of peripheral CPB	Vascular access complications; longer OR time	Early feasibility series [3,8,9]
Beating-Heart TECAB	Fully endoscopic (3–4 ports)	Endoscopic robotic LITA/BITA harvest	Robotic, endoscopic, automated connectors or hand-sewn	None required, optional pump-assist	1–4	Preserved EF, favorable target vessels	Steepest learning curve; conversion risk	Large single-center series (largest, *n* = 874) [13,24]
Staged HCR	LITA-LAD via RA-MIDCAB/TECAB + separate PCI session	As above	As above for surgical component; percutaneous stenting for PCI component	None required, optional pump-assist	1 surgical + PCI for non-LAD vessels	Multivessel disease with suitable PCI targets	Two-stage risk (interval ischemia, DAPT-related bleeding, or stent thrombosis); PCI incompleteness	RCTs available (HREVS, MERGING) plus observational series [25,26,27]
Simultaneous (“one-stop”) HCR	LITA-LAD + PCI same session, hybrid OR	As above	As above	None required, optional pump-assist	1 surgical + PCI for non-LAD vessels	Requires hybrid OR and coordinated team	Resource-intensive; radiation/contrast exposure	Observational series only [28]

**Table 2:** Principal Clinical Studies of Robotic Coronary Revascularization.

Study	Design	Procedure	*n*	Comparator	Centre Volume/Setting	Follow-Up	Patency Assessment (Method; Denominator)	Principal Outcome(s)	Key Limitation
Hwang et al. [1]	Meta-analysis (observational-dominated)	RA-MIDCAB, TECAB (pooled)	39 studies (21,642 patients)	Pooled comparisons vs. conventional CABG across included studies (heterogeneous)	Multiple centers; volume NR	Mean 5.2 years (pooled)	NR (pooled meta-analysis; not separately reported)	Conversion <3.2%; ~96% graft patency; 83–92% freedom from MACCE	Substantial heterogeneity across included studies; predominantly observational
Nisivaco et al. [24]	Single-center retrospective case series	Beating-heart TECAB	874	None (single-arm)	Single high-volume expert center	Mean ~48 months (longest 10.6 years)	NR (denominator not specified in primary report)	Perioperative mortality 0.9%; early graft patency 97%; freedom from MACCE 93%	Single-center, single-arm series; not yet replicated at other institutions
Jonsson et al. [17]	Single-center retrospective case series	RA-MIDCAB	1000	None (single-arm; learning curve focus)	Single high-volume center	NR (procedure time trend across cohort, not a survival follow-up duration)	Angiographic; 505/1000 patients assessed (50.5%)	30-day mortality 0.6%; graft patency 97.2% (*n* = 505 assessed); mastery required 250–500 cases	Angiographic assessment not obtained in full cohort; single-center
Kofler et al. [47]	Propensity-matched cohort	Robotic vs. conventional CABG	134 vs. 134	Conventional (sternotomy) CABG	Single center	Mean 6.6 years	NR	Statistically similar MACCE-free and overall survival between groups	Single-center; propensity-matched, not randomized; residual confounding possible
Liu et al. (TriNet X) [48]	Propensity-matched registry analysis	Robotic vs. non-robotic CABG	1796 vs. 249,580	Non-robotic CABG	Multi-institutional administrative database	NR (duration not specified in primary analysis)	NR	Similar long-term survival; lower stroke; higher repeat revascularization (robotic)	Administrative/claims data; no angiographic or anatomical detail
de Jong et al. [49]	Nationwide population-level registry	RA-MIDCAB, HCR	440 (91 HCR)	Population-level, non-randomized	Multiple centers nationwide, including non-academic	NR	NR	Robotic coronary surgery performed safely outside high-volume academic referral centers	Outcomes at lower-volume centers not separately reported in sufficient detail
Sardar et al. [50]	Meta-analysis (observational-dominated)	HCR	8 studies (2245 patients)	HCR vs. conventional CABG	Multiple centers; volume NR	Short-term reported; longer-term TVR follow-up duration NR	NR (pooled meta-analysis)	Lower transfusion/infection rates, shorter LOS, comparable short-term MACCE; higher long-term TVR	Heterogeneous HCR definitions; observational source studies
Nagraj et al. [51]	Meta-analysis (14 studies)	HCR	4226	HCR vs. conventional CABG	Multiple centers; volume NR	5 years	NR (pooled meta-analysis)	Statistically similar 5-year mortality and long-term MACCE; shorter stay, higher repeat revascularization (HCR)	Observational source studies; reporting heterogeneity
Pasrija et al. [52]	Retrospective cost comparison	TECAB vs. RA-MIDCAB	100 (50 vs. 50)	RA-MIDCAB (no conventional CABG arm)	Single center	Short-term (index hospitalization)	NR	TECAB had significantly higher hospital costs ($33,769 vs. $22,679) with comparable short-term clinical outcomes	No conventional CABG comparator arm; single-center; cost-focused
Patrick et al. (STS) [53]	Registry (STS database)	Robotic CABG (mixed)	1195 procedures/114 surgeons	None (single-arm; learning curve focus)	Multi-institutional (STS database)	NR (procedure-level, not longitudinal survival follow-up)	NR	Operative time and conversion rate stabilized after approximately the 10th case per surgeon	Reflects a minimum case volume competency threshold, not proficiency or mastery

**Table 3:** Randomized and Comparative Studies of Hybrid Coronary Revascularization.

Trial/Study	Design	*n*	Arms (Comparator)	Follow-Up	Primary Endpoint	Main Findings	Key Limitations
HREVS [26]	RCT	155	CABG vs. HCR vs. multivessel PCI	12 months	Residual myocardial ischemia (SPECT); MACCE	Non-inferior ischemia HCR vs. CABG (5% vs. 5%); similar MACCE (12% CABG, 13.4% HCR, 13.2% PCI, *p* = 0.83)	Single-center; short follow-up; SPECT surrogate endpoint
MERGING (pilot) [27]	RCT (pilot, 2:1 randomization)	60	HCR vs. CABG	Long-term (exact duration not specified in cited report)	Composite: death, MI, stroke, unplanned revascularization	19.3% HCR vs. 5.9% CABG, driven by unplanned revascularization	Small pilot sample; not powered for definitive conclusions
Balkhy et al. [63]	Propensity-matched, intent-to-treat	418	Complete HCR vs. incomplete (PCI omitted, ~20%)	Midterm (exact duration NR)	Midterm survival; MACE	No significant difference with PCI omission	Non-randomized; moderate sample; possible selection bias in PCI-failure subgroup
Sardar et al. [50]	Meta-analysis (observational-dominated)	Pooled (8 studies, 2245 patients)	HCR vs. CABG	Short-term reported; longer-term TVR follow-up duration NR	Transfusion, infection, LOS, MACCE, TVR	Lower transfusion/infection/LOS; comparable short-term MACCE; higher long-term TVR (HCR)	Heterogeneous HCR definitions; observational source studies
Nagraj et al. [51]	Meta-analysis (14 studies)	4226	HCR vs. CABG	5 years	5-year mortality; long-term MACCE	Similar 5-year mortality/MACCE; shorter stay, higher repeat revascularization (HCR)	Observational source studies; reporting heterogeneity
Schuering et al. [62]	Meta-analysis (32 studies)	2048	Conventional HCR vs. robot-assisted HCR	Perioperative (short-term)	Perioperative safety	Favorable perioperative safety profile for robotic HCR	Predominantly observational comparative data

**Table 4:** Principal Learning Curve Studies in Robotic Coronary Artery Bypass Grafting.

Study	Design	Procedure Studied	Cases/Surgeons (*n*)	Follow-Up/Data Period	Outcome/Endpoint Used	Key Finding
Patrick et al. (STS database) [53]	Registry (retrospective, multi-institutional)	Robotic CABG (mixed)	1195 cases/114 surgeons	NR (procedure-level analysis, not longitudinal)	Operative time, conversion—competency threshold	Stabilization after ~10th case per surgeon
Jonsson et al. (Emory) [17]	Single-center retrospective case series	RA-MIDCAB (single-vessel)	1000 consecutive cases, 1 center	NR (procedure-time trend, not survival follow-up)	Operative time, conversion rate, mortality, patency—mastery threshold	Mastery required 250–500 cases; conversion fell from 4.4% to 1.6%
Rosati et al. [65]	Single-center retrospective (new program)	RA-MIDCAB, new program	Single newly adopting center	NR	Safety during program initiation	Patient complexity did not adversely affect safety
Shekar et al. [59]	Single-center retrospective, CUSUM analysis	Robotic vs. conventional CABG	Single center, CUSUM analysis	NR	Postoperative atrial fibrillation; CUSUM proficiency curve	Proficiency suggested by 86th case (center-specific)

## Data Availability

No new data were created or analyzed in this study. Data sharing is not applicable to this article.
